# The temperament of pre-teens at risk of educational and social exclusion

**DOI:** 10.3389/fpsyg.2023.1173175

**Published:** 2023-06-14

**Authors:** Alina Daniela Moanţă, Florin Pelin, Corina Ciolcă, Marian Costin Nanu, Georgeta Mitrache, Dan Badea, Lucian Mihai Ciuntea

**Affiliations:** ^1^Faculty of Physical Education and Sport, National University of Physical Education and Sport, Bucharest, Romania; ^2^Faculty of Physical Education and Sport, University of Craiova, Craiova, Romania; ^3^Faculty of Movement, Sport and Health Sciences, Vasile Alecsandri University of Bacău, Bacău, Romania

**Keywords:** temperament, social exclusion, school dropout, pre-teens, pre-teens temperament

## Abstract

**Introduction:**

Vulnerability and poverty increase the educational and social exclusion of pre-teens. The goal of the present study was to identify the temperamental characteristics of pre-teens at risk of educational and social exclusion, depending on type of vulnerability and gender.

**Methods:**

For the study, 329 students (167 boys and 162 girls) at risk of early school leaving were involved and grouped into four categories: preadolescents from single-parent families, students with an absent parent (for example, is working abroad), socially assisted students, and Roma pre-teens (socially assisted). To assess temperament the Early Adolescent Temperament Questionnaire (EATQ-R) was used.

**Results:**

The results underline that in the case of the four super factors and for the two behavioral scales investigated, the scores (at group level) are, generally, within the average limits. The study highlights the importance of specialists to increase, in the case of pre-teens at risk of early school leaving, Effortful Control, and to decrease Negative Affectivity (which includes frustration and fear) and Depressive Mood. Significant differences between vulnerable boys and girls were observed, in the case of Surgency, Affiliation, and Depressive Mood. Also, using the Mann-Whitney (U) test and an independent sample *t*-test, gender-dependent differences were highlighted (considering the EATQ-R scales), in the case of each type of vulnerability. Using single-factor multivariate analysis of variances, the differences between preadolescents, depending on the type of vulnerability, were presented.

**Discussion:**

In the case of Surgency, boys registered significantly higher scores than girls, while in the case of Affiliation and Depressive Mood girls obtained higher values. Gender- and type of vulnerability-dependent differences in temperament were discussed in the case of pre-teens, and showed a temperament-conscious education is necessary in future parental education and teacher training.

## 1. Introduction

School dropout represents a considerable social problem in Romania, with the consequences observed for both individuals and society (Chalita et al., 2012). Educational and social exclusion was worsened by the COVID-19 pandemic, with poverty often making access to technology impossible. As Quílez-Robres et al. (2022) argued, the suspension of traditional school education (to minimize the infectious spread of the SARS-CoV-2 virus) “was a handicap for teaching due to the change in teacher-student relationships, but especially at early ages and in relation to the most disadvantaged classes (rural areas-low socioeconomic level).” Poor access to technology and preadolescents' vulnerabilities (for example innate or acquired learning difficulties, or high levels of stress and anxiety), together with older siblings' and parents' attitude regarding school and learning, represent variables which have a strong impact on the decision to drop out of school (Terry, 2008; Pelin et al., 2021). Parental education (an important pillar of society) has the potential to diminish/mitigate the negative impact of factors which can determine the social exclusion of a child, such as belonging to a single-parent family or living in poverty (Churchill and Clarke, 2009). Developing emotional intelligence in parents (and teachers) makes parenting (and teaching) practices more efficient (Kokkinos et al., 2021).

In 2021, the percentage of social exclusion in Romania was the highest across the European Union (EU) Member States (34%), with Bulgaria (32%) ranking second and Spain and Greece third, both at 28% (Eurostat, 2022). When talking about educational exclusion and school dropout in lower secondary education, over the last 20 years various studies have been carried out in Romania. For example, an increase in the number of students who dropped out of school before entering upper secondary education (high-school) was observed—the value almost tripled from 2000 to 2008 (Gyönös, 2011). Also, between 2008 and 2013, on average, only 83% of children enrolled in grade 1 (8 years ago) were still in school at the end of grade 8/lower secondary education (Ministry of National Education, 2018).

Since the 1980's researchers have examined the role of temperament in education, school adjustment, and academic achievement and presented them as essential variables investigated (Al-Hendawi, 2013). Considering middle school, it was found that students who dropped out (ages 11–13) were lethargic and casual in nature, reflective, and accepting, whereas those who continued with education were more practical, critical, active, premeditated, and responsible in nature (Singh, 1989). Teachers, parents, and peers, together with a child's academic self-concept, mediate the temperament effect on educational result (Martin, 1992). A negative temperment is linked to disruptive classroom behavior (in primary grade children), high maintenance students manifesting higher levels of overt aggression, attentional difficulties, emotional-oppositional behavior, and covert disruptive behavior (McClowry et al., 2013).

Researchers mention that school performance is influenced by the individual differences in pre-teens and adolescent's temperament (Valiente et al., 2007; Checa and Rueda, 2011). Academic failure also represents a major indicator of dropping out of school—low grades predict social and educational exclusion (Dockery, 2012). During preadolescence, temperament assessment is important to predict school adaptability, the risk for externalizing or internalizing problems and, also, the level of development of emotional and social abilities (Liu et al., 2011). Checa and Abundis-Gutierrez (2017) emphasized a positive correlation between temperament factor of regulation and school success and a negative link between temperament factor of reactivity and academic success. Following a meta-analysis, Poropat (2009) asserted that “extroverts have better school results due to their increased energy and positive attitudes” (features which are accompanied by a desire to assimilate and to understand the lessons), while negative emotions and low scores for emotional stability are related to low academic results and absenteeism.

As temperament (or dynamic-energetic subsystem of the personality) has a strong genetic determination, something which can be understood from an early period in a person's life (Kagan and Snidman, 1999; Rothbart, 2007), it is one of the components of personality which is investigated in primary selection. Temperament manifests itself in a person's behavior, being the most easily ascertainable component of the personality (Predoiu et al., 2021), affecting the intensive and mobility features at cognitive, affective, motor, and verbal levels (for example, voice intensity, rhythm, and speech tempo. However, temperament does not determine their quality—e.g., the persuasive force of the verbal message). It also refers to the biological and neuro-chemical properties of nervous systems (Sulis, 2018) and determines the internal way in which an individual reaches a given state (with a higher or lower energy consumption) and it changes throughout life, especially in terms of expression, as a result of maturation—for example, the explosive nature of the newborn is no longer manifested in the same way in adults (Mitrache and Predoiu, 2016). As Cyniak-Cieciura (2021) mentioned, temperament describes “the speed of learning new and unlearning emotional and behavioral reactions.”

Research on temperament in children has been dominated by two theoretical models. The first is based on research by Jerome Kagan and colleagues and has behavioral inhibition as its central concept. Behavioral reactions associated with behavioral inhibition include avoiding unfamiliarity, reducing the frequency of smiles and verbalizations, and increasing closeness to family members (Kagan et al., 1984; Reznick et al., 1986). Unlike the first model that operates with a dichotomy (inhibited temperament vs. uninhibited temperament), Rothbart (2007) proposed a three-factor model of temperament during childhood and adolescence, referring to Negative Affect, Surgency-Extraversion, and Effortful Control. It is worth mentioning that, recently, Lipska et al. (2022) discussed this “composition” of temperament when investigating Polish children. When talking about early adolescence, a fourth factor, called affiliativeness, was emphasized (Ellis and Rothbart, 2001; Putnam et al., 2001), involving a need and desire for closeness with others, and concern for others (Ellis and Rothbart, 2001). Rothbart discusses (in the psychobiological model developed) reactivity and self-regulation (Rothbart et al., 2000a). Reactivity refers to the Negative Affect and Surgency/Extraversion (level of activation), while, when talking about self-regulation, literature refers to Effortful Control (Putnam et al., 2006). Temperament is conceptualized as the self-regulation of attention-related processes and activities, while reactivity refers to the physiological excitability of neural systems, through self-regulating individuals are able to modulate this involuntary, automatic reactivity (Rothbart, 2011). As Rothbart and Rueda (2005) asserted, behavioral activation processes help individuals to sustain or to initiate a non-dominant response, while behavioral inhibition processes allow us to suppress a dominant response when needed.

The Negative Affect dimension includes frustration and fear, referring to a predisposition to experience negative emotions, including discomfort, dysphoria, and/or anger (see Hoffmann et al., 2017). Frustration can activate approach tendencies in relation to a new, unfamiliar situation, while fear implies a predisposition toward inhibitory tendencies. Inhibition has adaptive purposes in the sense that non-discriminatory approach tendencies to all stimuli can be dangerous (Rothbart and Bates, 1998). Extraversion/Surgency is the result of an increased sensitivity to rewards, refers to the level of activity, and is manifested by a tendency to explore new stimuli and situations (Rothbart and Bates, 2006); emotional reactivity is expressed by positive emotionality, and can be assessed in laboratory studies in children younger than one (Rothbart et al., 2000b). Effortful Control is a consequence of the development of executive attention functions and involves the ability to regulate emotional states by redirecting attentional resources, as well as the ability to suppress initial behaviors and reactions (that may interfere with personal goals), in order to adapt to the demands of the situation (Rothbart and Bates, 2006; Fitzpatrick et al., 2022). From the age of three, studies have shown that voluntary control skills remain relatively stable from preschool to adolescence (Kochanska et al., 2000).

The goal of the current research was 2-fold: to identify the temperamental characteristics of pre-teens at risk of educational and social exclusion, depending on type of vulnerability, and to underline gender differences in vulnerable preadolescents (considering temperament). Four groups were formed: students with an absent parent (for example, is working abroad), socially assisted students, Roma preadolescents (socially assisted), and pre-teens from single-parent families.

The following research questions were asked:

1. What are the temperamental features of pre-teens at risk of educational and social exclusion depending on type of vulnerability?
2. What are the gender-dependent differences in vulnerable preadolescents, when talking about temperament?

## 2. Materials and method

### 2.1. Participants

Three hundred and twenty-nine students (167 boys and 162 girls) at risk of educational and social exclusion were involved in the current study. They were students in grades V-VII (between 11 and 13 years old, *M*_Age_ = 12.4) at different schools, from various regions of Romania: Bacău county, Braşov county, Bucharest, Buzău county, Timiş, Neamţ, and Dâmboviţa county.

Of the 329 students, 38 had an absent parent (is working abroad; 22 boys and 16 girls), 184 were socially assisted pre-teens (87 boys and 97 girls), 70 were Roma preadolescents (socially assisted; 39 boys and 31 girls), and 37 were pre-teens from single-parent family (19 boys and 18 girls).

### 2.2. Measures

The longer version of the Early Adolescent Temperament Questionnaire—revised form (EATQ-R)—was used in the present research. EATQ-R was developed starting from Rothbart theory (Ellis and Rothbart, 2001; Viñas et al., 2015) and was calibrated for the Romanian population, being part of the PEDb computerized platform developed by Cognitrom (Miclea et al., 2012).

The 11 temperament scales of the EATQ-R are distributed according to four super factors: three scales belong to the Effortful Control super factor—Attention (e.g., *I find it easy to concentrate on my homework*); Inhibitory Control (the capacity to suppress inappropriate verbal and motor responses)—e.g., *When I'm told to stop doing something, it's easy for me to stop*; and Activation Control (the capacity to carry out a task even if there is a strong tendency to avoid it)—e.g., *When someone asks me to do something, I do it immediately, even if I don't really want to*. Two scales relate to the Surgency super factor—Activation level (engaging in activities involving high levels of physical activity)—e.g., *I'd rather play a sport than watch TV*; and High Intensity Pleasure (the pleasure generated by activities involving high intensity or novelty)—e.g., *I would get excited about the idea of driving a race car*. Two scales belong to the Negative Affectivity super factor—Frustration (e.g., *It annoys me when I try to make a call and it rings busy*) and Fear (e.g., *I worry about getting into trouble*), while 4 scales belong to the Affiliation super factor—Affiliation (the need for closeness with others)—e.g., *I want to be able to share my personal thoughts with someone else*; Shyness (e.g., *I'm shy about meeting new people*); Pleasure Sensitivity (pleasure determined by activities involving low intensity, complexity, or novelty)—e.g., *I like to look at the trees and walk among them*); and Perceptual Sensitivity (ability to identify low-intensity stimuli in the environment)—(e.g., *I tend to notice small changes that others don't notice)*. EATQ-R also assesses two behavioral scales: Aggression (e.g., *If I am angry with someone I tend to say things that I know will hurt them*) and Depressive Mood (e.g., *I feel like crying very easily*). The questionnaire consists of 103 items (and is a self-report measure).

The participants responded using a 5-step Likert scale, where 5 = always true; 4 = usually true; 3 = sometimes true, sometimes false; 2 = usually false; and 1 = always false. In the current study, internal consistency estimates (McDonald's omega coefficient—ω) ranged from 0.72 to 0.80 for the behavioral scales (aggression and depressive mood) and the four super factors.

### 2.3. Procedure

The participants completed the EATQ-R (Early Adolescent Temperament Questionnaire) between June 2021 and September 2022. The questionnaire was applied following direct contact with pre-teens in the camps organized within the project entitled “Sustainable social and educational integration through sports activities—PNP001 2019–2023” (the camps took place at the seaside and in the mountains in Romania), and at the schools involved in the project. EATQ-R was applied in groups of 7–22 preadolescents (eligible for the project carried out).

In the current study, the purposive sampling technique was used, and preadolescents at risk of educational and social exclusion were investigated (socially assisted, from single-parent families, with a parent working abroad, or Roma preadolescents—socially assisted). Informed consent from the children's parents was obtained.

### 2.4. Statistical analysis

Through a single-factor multivariate analysis of variance, children's temperament was investigated depending on type of vulnerability. In the case of the Levene's test (homogeneity of variance), when *p* < 0.05 the Tamhane *post-hoc* test was interpreted, and when the *p*-values were insignificant the Scheffe *post-hoc* test was reported (Popa, 2010). SPSS 20 was used. Also, using jamovi program (The Jamovi Project, 2020), the reliability of the scales was calculated (McDonald's omega coefficients—ω). Independent sample *t*-test and Mann-Whitney *U*-test were also used. Normality condition (in the case of *t*-test) was checked through the skewness scores, with the absolute values being < 1 (Morgan et al., 2004). The effect size index—Hedges's g was interpreted as follows: 0.2 small effect, 0.5 moderate, and 0.8 strong effect size (Predoiu, 2020). In the case of r (for Mann-Whitney U test), the values are: 0.1—small effect, 0.3—moderate, 0.5—strong effect (Ellis, 2010).

## 3. Results

First, we present the scores in the case of the four super factors of temperament (Effortful Control, Surgency, Negative Affectivity, and Affiliation) and the behavioral scales (Aggression and Depressive Mood) measured through EATQ-R according to the type of vulnerability (Table 1). The values are expressed in T scores, automatically generated, where 61–100 represents a high score, 45–55 represents an average score, and 0–39 represents a low score, 56–60 is slightly above average, while 40–44 is slightly below average (using the T scores gender and age variations were eliminated).

**Table 1:** Descriptive statistics—preadolescents at risk of educational and social exclusion.

**EATQ-R**	**Socially assisted pre-teens (*****n*** = **184)**		
	***M***	***SD***	***SE***
1	50.73	8.24	0.60
2	50.83	7.21	0.53
3	52.21	8.21	0.60
4	52.24	6.19	0.45
5	50.06	10.69	0.78
6	53.59	11.55	0.85
	**A parent is working abroad (*****n*** = **38)**		
1	49.99	6.54	1.06
2	53.27	4.85	0.78
3	52.21	7.96	1.29
4	56.50	5.87	0.95
5	48.68	8.41	1.36
6	53.13	7.14	1.15
	**Pre-teens from single-parent families (*****n*** = **37)**		
1	51.64	9.28	1.53
2	54.94	9.25	1.52
3	55.06	8.36	1.37
4	54.73	5.71	0.93
5	52.38	11.84	1.94
6	55.11	11.23	1.84
	**Roma preadolescents—socially assisted (*****n*** = **70)**		
1	47.81	5.22	0.62
2	51.29	6.28	0.75
3	54.87	6.90	0.82
4	52.01	6.53	0.78
5	54.33	8.85	1.05
6	56.06	11.24	1.34

1: Effortful Control; 2: Surgency; 3: Negative Affectivity; 4: Affiliation; 5: Aggression; 6: Depressive Mood.

The results presented above underline that, in the case of the four super factors and for the behavioral scales, the values (at group level) are, generally, within the average limits (45–55); only in some cases (depressive mood and affiliation) are the scores slightly above average (55–60). We mention the importance of specialists to increase, in the case of pre-teens at risk of early school leaving, Effortful Control (includes inhibitory control and attention), and to decrease Negative Affectivity (including frustration and fear) and Depressive Mood.

In a second phase, we investigated whether there are significant differences between girls and boys considering the six dependent variables (DVs) regardless of the type of vulnerability. *T*-test was used for independent samples (Table 2). The values for the alpha significance threshold, in the case of the Levene test, were insignificant—*p* > 0.05 (except for Effortful Control). The skewness values were < 1 (the absolute values), meaning the condition of normality was ensured.

**Table 2:** Vulnerable pre-teens - boys (*n* = 167) vs. girls (*n* = 162).

**EATQ-R**	***t***	***P***	**95% confidence interval**	
			**Lower**	**Upper**
Effortful control	−1.465	0.144	−2.925	0.428
Surgency	4.388	0.000	1.862	4.889
Negative affectivity	−0.833	0.405	−2.473	1.001
Affiliation	−3.465	0.001	−3.735	−1.029
Aggression	0.322	0.747	−1.883	2.620
Depressive mood	−2.961	0.004	−5.877	−1.142

It can be observed that there are significant differences between vulnerable boys and girls in the case of Surgency, Affiliation, and Depressive Mood. In the case of Surgency, boys registered higher scores than girls (*M*_boys_ = 53.32, *SD* = 7.35; *M*_girls_ = 49.95, *SD* = 6.56), while in the case of Affiliation and Depressive Mood girls obtained higher values (*M*_girlsAffiliation_ = 54.17, *SD* = 5.92; *M*_boysAffiliation_ = 51.79; *SD* = 6.52; *M*_girlsDepressiveMood_ = 56.01, *SD* = 11.66; *M*_boysDepressiveMood_ = 52.50, *SD* = 10.12). The effect size is g = 0.48 (Surgency), g = 0.38 (Affiliation), and g = 0.32 (Depressive Mood).

In order to have a more detailed picture of the existing differences between boys and girls at risk of educational and social exclusion (considering temperament), the individual scales of EATQ-R were also examined. Various types of vulnerability were taken into account: pre-teens from single-parent family (M group), pre-teens with a parent working abroad (PP group), socially assisted preadolescents (AS), and Roma preadolescents—socially assisted (R). The non-parametric test Mann-Whitney (U) was used for M and PP groups (considering the reduced sample size), and *t*-test for independent samples was used for AS and R groups. Normality (in the case of *t*-test) was checked through the skewness scores (the absolute values were < 1).

Analyzing Table 3, significant differences were highlighted between boys and girls (preadolescents from single-parent families) when talking about Fear and Aggression. Boys registered lower values than girls for both Fear and Aggression—Fear (*M*_boys_ = 52.13, *SD* = 8.61; *M*_girls_ = 61.40, *SD* = 10.16; *p* = 0.039) and Aggression (*M*_boys_ = 47.54, *SD* = 8.91; *M*_girls_ = 57.28, *SD* = 12.78; *p* = 0.014).

**Table 3:** *U*-test - pre-teens (boys vs. girls) according to the type of vulnerability.

**EATQ-R scales**	**Pre-teens from single-parent families boys (*****n*** = **19) and girls (*****n*** = **18)**		
	***Z***	***P***	***r***
AC	−1.218	0.223	0.200
AL	−1.111	0.267	0.182
Attention	−0.529	0.597	0.086
Affiliation	−1.033	0.302	0.169
Frustration	−0.902	0.367	0.148
Fear	−2.065	0.039	0.34
IC	−1.008	0.314	0.165
HIP	−1.403	0.161	0.230
PS	−0.106	0.916	0.017
PERS	−1.166	0.244	0.191
Shyness	−1.716	0.086	0.282
Aggression	−2.419	0.014	0.40
DM	−1.568	0.118	0.257
**EATQ-R scales**	**A parent is working abroad boys (*****n*** = **22) and girls (*****n*** = **16)**		
	***Z***	***P***	***r***
AC	−1.977	0.048	0.32
AL	−0.566	0.572	0.091
Attention	−0.587	0.557	0.095
Affiliation	−1.331	0.183	0.215
Frustration	−0.779	0.436	0.126
Fear	−2.926	0.003	0.47
IC	−1.341	0.180	0.174
HIP	−0.894	0.371	0.145
PS	−0.189	0.850	0.030
PERS	−2.304	0.021	0.37
Shyness	−0.730	0.465	0.118
Aggression	−0.711	0.492	0.115
DM	−2.109	0.036	0.34

AC, Activation Control; AL, Activation level; IC, Inhibitory Control; HIP, High Intensity Pleasure; PS, Pleasure Sensitivity; PERS, Perceptual Sensitivity; DM, Depressive Mood.

Regarding the effect size index, *r* = 0.34 (Fear) and *r* = 0.40 (Aggression). Thus, the impact of the group variable on the results for Fear and Aggression is moderate to strong.

Significant differences were found between boys and girls (PP group) in the case of Activation Control (*M*_boys_ = 52.95, *SD* = 6.46; *M*_girls_ = 58.00, *SD* = 11.57; *p* = 0.048), Fear (*M*_boys_ = 51.05, *SD* = 5.94; *M*_girls_ = 59.88, *SD* = 6.87; *p* = 0.003), Perceptual Sensitivity (*M*_boys_ = 49.80, *SD* = 9.27; *M*_girls_ = 57.38, *SD* = 7.17; *p* = 0.021), and Depressive Mood (*M*_boys_ = 51.05, *SD* = 5.94; *M*_girls_ = 56.00, *SD* = 7.82; *p* = 0.036).

Considering the effect size, *r* = 0.32 (Activation Control), *r* = 0.47 (Fear), *r* = 0.37 (Perceptual Sensitivity), and *r* = 0.34 (Depressive Mood). In other words, the impact of the group variable (girls or boys, from PP group) on the scores for Activation Control, Fear, Perceptual Sensitivity, and Depressive Mood, is moderate or moderate to strong (Predoiu, 2020).

Considering socially assisted preadolescents, according to Table 4, the mean values for Activation Control and Activation level scales are significantly higher (*p* = 0.015, respectively *p* = 0.008) in girls (Activation Control: *M*_girls_ = 55.75, *SD* = 10.61; Activation level: *M*_girls_ = 53.20, *SD* = 8.25) compared to boys (Activation Control: *M*_boys_ = 52.01, *SD* = 9.80; Activation level: *M*_boys_ = 49.02, *SD* = 11.85). The Hedge's *g*-value was calculated (effect size)—*g* = 0.36 (Activation Control), *g* = 41 (Activation level). Therefore, there is a moderate to weak difference between the results. A significant difference between boys (*M*_boys_ = 52.87, *SD* = 8.02) and girls (*M*_girls_ = 47.70, *SD* = 8.03) when talking about Shyness (*p* = 0.000) was also highlighted, *g* = 0.64, which underlines a moderate to strong difference between the scores obtained for Shyness by socially assisted boys and girls.

**Table 4:** *T* test - pre-teens (boys vs. girls) socially assisted (*n* = 87 boys, *n* = 97 girls) and Roma preadolescents—socially assisted (*n* = 39 boys, *n* = 31 girls).

**EATQ-R scales**	***t***		***P***		**95% confidence interval**			
					**Lower**		**Upper**	
	**AS**	**R**	**AS**	**R**	**AS**	**R**	**AS**	**R**
AC	−2.450	0.464	0.015	0.645	−6.749	−3.279	−0.727	5.260
AL	−2.691	−1.297	0.008	0.199	−7.236	−8.287	−1.113	1.764
Attention	1.145	0.535	0.254	0.595	−1.328	−3.154	4.998	5.458
Affiliation	0.906	2.564	0.366	0.013	−1.521	1.259	4.104	10.150
Frustration	−0.681	0.615	0.497	0.541	−3.935	−2.369	1.917	4.474
Fear	−1.643	−0.952	0.102	0.345	−6.230	−8.413	0.569	2.985
IC	0.116	−0.329	0.908	0.743	−2.546	−5.085	2.865	3.647
HIP	0.545	1.255	0.586	0.214	−2.096	−1.293	3.696	5.655
PS	0.642	−1.979	0.522	0.052	−2.084	−8.614	4.094	0.043
PERS	−1.219	0.199	0.224	0.843	−4.513	−3.781	1.067	4.619
Shyness	4.329	0.460	0.000	0.647	2.815	−3.205	7.530	5.119
Aggression	1.116	0.302	0.266	0.764	−1.353	−3.630	1.605	4.926
DM	−1.703	−1.164	0.090	0.249	−6.168	−8.524	2.599	2.244

AS, socially assisted; R, Roma preadolescents (socially assisted); AC, Activation Control; AL, Activation level; IC, Inhibitory Control; HIP, High Intensity Pleasure; PS, Pleasure Sensitivity; PERS, Perceptual Sensitivity; DM, Depressive Mood.

In the case of Roma preadolescents (socially assisted), the mean result for Affiliation is higher (*p* = 0.013) in boys (*M*_boys_ = 54.17, *SD* = 10.15) compared to girls (*M*_girls_ = 48.47, *SD* = 7.26). The Hedge's *g*-value is 0.63 (there is a moderate to strong difference between the scores obtained by the investigated groups). Also, the mean result for Pleasure Sensitivity is lower (*p* = 0.052—a marginal significant difference can be observed) in boys (*M*_boys_ = 54.11, *SD* = 8.88) compared to girls (*M*_girls_ = 58.40, *SD* = 8.48). The Hedge's *g*-value is 0.49 (a moderate difference is emphasized); the upper limit is 0.043, whereas the lower limit is −8.614.

Also, using single-factor multivariate analysis of variances we verified the existing differences considering temperamental features in preadolescents depending on the type of vulnerability. SPSS 20 and type I procedure was used (for MANOVA).

There are weak correlations between the six DVs (Effortful Control, Negative Affectivity, Surgency, Affiliation, Aggression, and Depressive Mood), with linearity assumption being emphasized. Investigating the Box *M*-test—*p* = 0.000, therefore, we referred to the Pillai's Trace test value. Partial Eta Squared result (η^2^ = 0.05) indicates a moderate effect size (Predoiu, 2020), while the Observed Power is very high (>0.996). The confidence considering the stability of the results is strong. When homogeneity of variance was assured, we interpreted the Scheffe test and when *p* < 0.05 (Levene's test) we interpreted the Tamhane test.

Table 5 comprises only the significant differences between the investigated groups (pre-teens from single-parent family—M group, pre-teens in which case a parent is working abroad—PP group, socially assisted—AS group, Roma preadolescents—R), in terms of the six DVs examined.

**Table 5:** *Post-hoc* tests—one-way MANOVA (*n* = 184—AS group, *n* = 38—PP group, *n* = 70—R group, and *n* = 37—M group).

**Dependent variables**		**(I) Vulnerability**	**(J) Vulnerability**	***p***	**95% confidence interval**	
					**Lower bound**	**Upper bound**
Effortful control	Tamhane (Levene's test: *F* = 5.477, *p* = 0.001)	AS	PP	0.991	−2.571	4.067
			M	0.995	−5.419	3.600
			R	0.006	0.604	5.235
		PP	AS	0.991	−4.0.67	2.571
			M	0.941	−6.704	3.389
			R	0.403	−1.171	5.515
		M	AS	0.995	−3.600	5.419
			PP	0.941	−3.389	6.704
			R	0.138	−0.695	8.355
		R	AS	0.006	−5.235	−0.604
			PP	0.403	−5.515	1.171
			M	0.138	−8.355	0.695
Affiliation	Scheffe (Levene's test: *F* = 0.193, *p* = 0.901)	AS	PP	0.002	−7.3551	−1.1612
			M	0.173	−5.6261	0.6368
			R	0.995	−2.2096	2.6719
		PP	AS	0.002	1.1612	7.3551
			M	0.677	−2.2507	5.7777
			R	0.005	0.9872	7.9914
		M	AS	0.173	−0.6368	5.6261
			PP	0.677	−5.7777	2.2507
			R	0.197	−0.8069	6.2584
		R	AS	0.995	−2.6719	2.2096
			PP	0.005	−7.9914	−0.9872
			M	0.197	−6.2584	0.8069
Aggression	Tamhane (Levene's test: *F* = 3.172, *p* = 0.024)	AS	PP	0.946	−2.90	5.65
			M	0.855	−8.08	3.44
			R	0.009	−7.79	−0.75
		PP	AS	0.946	−5.65	2.90
			M	0.552	−10.15	2.76
			R	0.010	−10.30	−0.99
		M	AS	0.855	−3.44	8.08
			PP	0.552	−2.76	10.15
			R	0.945	−7.99	4.09
		R	AS	0.009	0.75	7.79
			PP	0.010	0.99	10.30
			M	0.945	−4.09	7.99

AS, socially assisted students; PP, students in which case a parent is missing (is working abroad); R, Roma preadolescents (socially assisted); M, preadolescents from single-parent families.

After running *post-hoc* tests (see Table 5), the following significant differences were emphasized:

- for Effortful Control (*p* = 0.006), between socially assisted pre-teens (*M*_sociallyassisted_ = 50.73) and Roma preadolescents (socially assisted)—*M*_Romapreadolescents_ = 47.81;
- for Affiliation, between students with a parent working abroad—*M*_aparentisworkingabroad_ = 56.50 and socially assisted preadolescents (*p* = 0.002, *M*_sociallyassisted_ = 52.24) and Roma pre-teens—socially assisted (*p* = 0.005, *M*_Romapre − teens_ = 52.01);
- for Aggression, between Roma preadolescents (socially assisted) and socially assisted students (*p* = 0.009) and pre-teens with a parent working abroad (*p* = 0.010)—*M*_Romapreadolescents_ = 54.33; *M*_sociallyassisted_ = 50.06; M_aparentisworkingabroad_ = 48.68.

## 4. Discussion

Considered the precursor of personality (Rothbart, 2007), temperament reflects the inter-individual differences in physiological and emotional reactions linked with the level of mental arousal and self-regulation (Rothbart et al., 2000a). Even if temperamental characteristics are relatively stable, as a result of children's learning experiences, certain aspects can be modeled (Kagan et al., 2007).

Understanding temperament is very important in order to promote school performance and adjustment (Checa and Abundis-Gutierrez, 2017). Also, research into temperament in children and adolescents is of great relevance, leading to comprehension of children's communication and interactions with others (Viñas et al., 2015). A particularly important problem refers developing positive (desirable) behaviors in pre-teens and adolescents and suppressing inappropriate ones (Pelin et al., 2018). It seems that a lower level of emotional reactivity (as a temperament trait) is associated with a good level of self-control (Necka et al., 2019), an important component of academic success, while “negative emotionality is associated with high psychological and behavioral controlling attempts of mothers” (Laukkanen et al., 2014).

In the case of preadolescents at risk of educational and social exclusion (in the current study), the following recommendations are made: teachers, psychologists, and parents can increase Effortful Control (the ability to regulate emotional states and to suppress initial reactions in order to adapt to the demands of the situation, also referring to concentration and mobility of attention) and decrease Negative Affectivity (which includes frustration and fear) and Depressive Mood. Systematic practice of physical activity can help in this matter. Greeff et al. (2017), following a meta-analysis, underlined that physical activity has positive effects on attention and academic performance in pre-teens. Physical activity plays an essential role in children's lives as it positively influences their health, both physically and mentally (Biddle and Asare, 2011), and also contributes fundamentally to their social, emotional, and cognitive development (Ahn et al., 2018). It is known that exercising regularly can reduce stress and improve mood, school performance, memory, and creativity in children (Goodliff et al., 2018; Mattke, 2019). Physical activity can increase children's enthusiasm, optimism, self-esteem, and help regulate behavior (Limpo and Tadrist, 2021). It also reduces anxiety, tension, and depression (Chavan, 2019). Motor activities, practiced on a group level, can stimulate teamwork, cooperation, communication, friendship and, therefore, social integration—especially when children regularly play sports (Wiese-Bjornstal et al., 2009; Pelin et al., 2021). Researchers argued for the beneficial effects of physical education activities at motor, structural-functional, and psychological levels (see Pelin et al., 2020). Aslan et al. (2020) asserted that “the attention levels of primary school students doing sports were better than those who were not doing sports,” recommending that children should be involved in physical activities, which decreases learning difficulty.

Statistical data processing highlighted that, in the case of Surgency, boys obtained significantly higher scores than girls, while in the case of Affiliation (the need for closeness with others) and Depressive Mood girls registered higher values. Thus, specialists can involve girls (pre-teens) to a greater extent in physical activities, in new and complex tasks involving collaboration and communication with others, and can teach preadolescents positive reframing/reappraisal, as this technique is associated with a reduced level of depression (Beck and Strong, 1982; Swoboda et al., 1990).

Considering the type of vulnerability:

- Socially assisted pre-teens have a significantly better ability to regulate emotional states by redirecting attentional resources, as well as a significantly better capacity to suppress initial reactions for better adaptation to the environment, compared to Roma preadolescents (socially assisted).
- Students with an absent parent (for example, is working abroad) have a significantly higher need for warm and close relationships with others compared to socially assisted pre-teens.
- Roma preadolescents have a significantly higher score for aggression (verbal aggression, hostility, and object- and person-directed physical violence) compared to both socially assisted students and preadolescents with a parent working abroad. It should be noted that the results (at group level) are within average limits.

When talking about gender-dependent differences, statistical data processing showed that boys (Roma preadolescents—socially assisted) feel a greater need for closeness to others, and also feel less pleasure in activities involving low intensity, complexity, or novelty compared to girls. With respect to socially assisted preadolescents (AS group), Activation level is lower in boys than in girls, while boys are significantly more shy than girls. When talking about Fear (PP and M groups), girls obtained higher scores than boys. In the case of pre-teens from single-parent families, girls registered a higher level of Aggression than boys, while in the case of pre-teens with a parent working abroad (is missing), girls obtained a significantly higher score for Depressive Mood. Specialists can thus intervene with priority in the case of girls (from PP and M groups) in order to reduce depressive mood and fear, and in boys (from AS group) for a higher activation level. In the case of children with low activity levels, researchers asserted that the benefits are greater when parents encourage their children to explore new stimuli (Blandon et al., 2010). Considering depressive mode in middle school students (6 grade), Moritz Rudasill et al. (2014) highlighted that depression symptoms in preadolescents were predicted by emotional reactivity (while negative emotionality predicted emotional reactivity), teachers' perceptions of student-teacher relationship quality, and pre-teens' perceptions of teacher support. The risk for depressive symptoms in children is higher, also, when parents use an authoritarian parenting style, based on discipline and strict punishments, at the expense of democratic discussions (Liu et al., 2022).

The way in which a professor, a psychologist, or a parent interacts with and motivates pre-teens and adolescents and guides them verbally or behaviorally plays a decisive role in student's success—in various activities (Lagace-Seguin and Coplan, 2005; Mitrache et al., 2018; Predoiu et al., 2020; Predoiu and Predoiu, 2022). Shi and Campione-Barr (2021) discuss the similarities between the parenting style and siblings' temperamental features. It seems that “a higher level of parenting similarity was related to more positive family relationships when siblings were more similar in their temperaments.” However, in the context of less sibling temperament similarity, a lower level of parenting similarity is preferable. Recent studies highlight the effect of screen time intake in child development, more specifically, screen media use (an exceedingly frequent activity) being detrimental to the development of Effortful Control (including attention, the ability to suppress inadequately answers—verbal or motor), and thereby shaping young children's temperaments (Fitzpatrick et al., 2022). Also, researchers found that parental monitoring acts as a protective factor for early substance use, especially in the case of more aggressive youth and those with low Effortful Control (Clark et al., 2015).

Recent specialized literature discusses the link between the levels of externalizing problems and temperamental factors (Surgency/Extraversion, Effortful Control, and Negative Affectivity), with maternal psychological distress partly explaining this link (see Garon-Carrier et al., 2022). As Liu et al. (2011) mention, internalizing behavior reflects a child's psychological or emotional state and typically includes depressive and anxious symptoms, social withdrawal, somatic complaints, and even teenage suicide. Externalizing behaviors on the other hand are described as aggressive, delinquent behaviors, being reflected by actions toward the physical environment (Eisenberg et al., 2001). Therefore, as Mullola et al. (2014) highlighted, “a temperament-conscious education needs to be taken into account in future teacher training” (and also in parental education, as an important pillar of society), early manifestations of self-control being linked to consequential life outcomes (Clark et al., 2015). The current research fills a gap in the literature considering the temperamental characteristics (according to vulnerability and gender) of pre-teens at risk of educational and social exclusion.

This research has some limitations. The first limitation is the reduced number of pre-teens from PP and M groups (pre-teens with a parent working abroad and from single-parent families). Also, the time a parent spent abroad was not investigated (in the case of PP group). The results could be different if other categories of students were investigated (for example, adolescents), if the research was carried out in another country, or if other temperamental typologies were approached (Jung and Eysenck typology, Pavlov's types of nervous system, Hippocrates-Galen temperaments, constitutional typologies, etc.). In the current research, an explicit tool was used, the possible effect (possible socially desirable responses) being emphasized by the literature (Predoiu et al., 2022). The large number of respondents represents, however, a strength of the study. Future studies could focus on children in a certain grade (5th, 6th, etc.), on children from a specific region in Romania, on children with special educational needs (having disabilities or learning difficulties), or could examine different factors of temperament in relation to academic performance.

## 5. Conclusion

The conclusions of the current study, carried out in various regions of Romania, show that in the case of the four super factors of temperament and for the behavioral scales, the values (at group level) are, generally, within the average limits. In the case of pre-teens at risk of educational and social exclusion, specialists should increase Effortful Control (which includes inhibitory control and attention) and decrease Negative Affectivity (which includes frustration and fear) and Depressive Mood. In the case of Surgency, boys registered significantly higher scores than girls, while in the case of Affiliation and Depressive Mood girls obtained higher values. Gender-dependent differences in temperament were presented in the case of pre-teens from single-parent families (M group), with a parent working abroad (PP group), socially assisted preadolescents (AS), and Roma preadolescents—socially assisted (R). Also, differences considering temperamental features in preadolescents were highlighted, depending on the type of vulnerability (M, PP, AS, and R). The particularly important role of systematic physical activity is underlined, having the ability to reduce depression, improve mood, and help regulate behavior.

## Data availability statement

The raw data supporting the conclusions of this article will be made available by the authors, without undue reservation.

## Ethics statement

The present study was approved by the Local Ethics Committee of the National University of Physical Education and Sport, Bucharest, authorization number assigned is ID: 187. Written informed consent to participate in this study was provided by the participants' legal guardian/next of kin.

## Author contributions

All authors listed have made a substantial, direct, and intellectual contribution to the work and approved it for publication.
